# Evolution of concentration and phase structure of colloidal suspensions in a two-ends-open tube during drying process

**DOI:** 10.1038/s41598-020-65879-0

**Published:** 2020-06-03

**Authors:** Shenwei Wang, Hongwei Zhou, Zhiwei Sun, Shenghua Xu, Wenze Ouyang, Linwei Wang

**Affiliations:** 1 Key laboratory of Microgravity (National Microgravity laboratory), Institute of Mechanics, Chinese Academy of Sciences, Beijing, 100190 China; 2 School of Engineering Science, University of Chinese Academy of Sciences, Beijing, 100049 China

**Keywords:** Chemical physics, Condensed-matter physics

## Abstract

We investigated the evolution of concentration and phase structure of colloidal suspensions in a two-ends-open tube during drying process. The volume fraction and crystal structure of suspension in the capillary tube were determined by reflection spectrometer during drying process. Our experimental results show: (a) evaporation takes place in two directions of the tube, though much stronger in one direction than the other; (b) during drying process, colloidal suspension column along the tube can be divided into four regions, namely, the close packed region, concentrated region, initial concentration region and dilution region. A new model describing the evolution of concentration profile was proposed and the calculated results based on the model are in good agreement with the experimental ones. According to solute conservation, we also present a simple way to estimate the concentration of close packed region.

## Introduction

Drying, as a common process in nature, has been widely utilized in many research and industry fields, such as printing, coating and ceramics^1–3^. In colloidal suspension, the drying process can also be used to induce self-assembly and form three-dimensional organized colloidal structures (colloidal crystals) through drying of droplets^4–6^, of films^7–10^ or in capillary tubes^11,12^. Among most of the methods above, a high concentration of colloids is reached locally (e.g., close to the drying interface of capillary tube) due to the interplay of evaporation and capillarity, forming the three-dimensional colloidal crystals. Especially, the drying colloidal suspension confined in capillary tube with two ends open offers an alternative approach to investigate drying process and evaporation phenomena^13,14^.

Drying process in capillary tube is of certain importance in many fields^15–19^. Through drying colloidal suspension in the tube, the acquired patterned surface can be used as a model for molding regular micro-/nanostructure and the rough surface can capture cells more efficient than blank glass surface which can provide promising application for biomedical areas^20^. Formation of basalt columns during cooling of lava can be modeled by the drying of colloidal suspension confined in capillary tube which shed new light on the basalt column formations^21,22^. Macroscopic soft colloidal crystals with fiber symmetry can be fabricated by drying colloidal suspension inside two end open tube with potential applications in photonic band gap materials, optical filters, sensors, and so forth^11^. Since the drying process in capillary tube is vital, the model for the concentration distribution inside the tube during this one-dimensional drying has also garnered attention^23,24^. For drying the colloidal suspension in capillary tube, the movement of suspension is normally in one direction and relevant models were developed to analyze the volume fractions of close packed structures or dense states^25^. By our understanding, the temporal concentration distribution should be essential experimental data for modeling because the process in the solidification involves significant mass transfer. However, it is difficult to measure the concentration distribution in real time along a slender capillary tube, especially for disordered suspension. We gather that lack of data for the concentration distribution should be the main reason why the previous models could not make prediction that is well consistent with experiments.

In this study we adopted a reflection spectrometer combined with a specially developed data-handling method^26^ to detect, in a non-invasive, real-time and *in-situ* manner, the volume fraction and crystal structure of suspension in the capillary tube. The measurement method relies on Bragg diffraction peak from crystal lattice structures. The wavelength of the peak is directly related to the distance between the particles and therefore, the local volume fraction of suspension, the key parameter in the study, can be estimated. Apparently, this method is valid only for ordered suspension. The relevant previous study related to the above topic has indicated that the movement of the suspension due to evaporation always started from one side of the tube and also the suspension was thought to be divided into two regions^25,27,28^. Distinguishing with these results, our study shows: (1) evaporation takes place in two directions of the tube, though much stronger in one direction than the other; (2) during drying process, colloidal suspension column along the tube can be divided into four regions, namely, the close packed region, concentrated region, initial concentration region and dilution region. We further put forward a new model to explain the evolution of concentration profile, the calculated results based on our model are in good agreement with the experimental results. According to solute conservation, we further suggest a simple way to estimate the concentration of close packed region.

## Results and Discussion

Since the reflection spectrum method used in this study is only applicable to the ordered suspensions, for ease of understanding, we will discuss suspension A (initially ordered suspension), and then B (initially disordered suspension).

### Drying process of initially ordered suspension (A)

**Figure 1:**
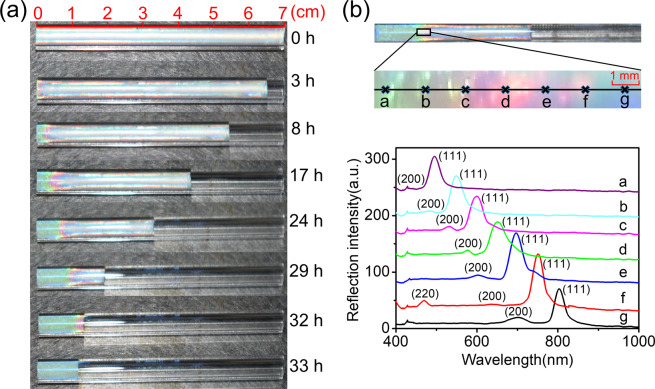
(**a**) Photographs of initially ordered suspension during drying process in chronological order. (**b**) Enlarged graph of the concentrated region with an approximate size $9\times 1\,m{m}^{2}$ (*a*-*g* represents the measure points with equal distance) and the corresponding reflection spectra (The spectra were shifted for clarity).

A series of photographs of initially ordered suspension (A) taken in chronological order are presented in Fig. 1(a). It can be observed that the movement of the colloidal suspension caused by evaporation always started from one side of the tube, although both sides of the tube are open. Intuitively, water should evaporate simultaneously from two sides because the tubes are horizontal and the environment for each side is the same. However, due to thermal fluctuations^11^ at some moment evaporation at one end of the tube may occasionally go exceptionally faster than at the other. This kind of evaporation stronger randomly at one end may firstly accumulate particles at the drying interface near this end, forming nanometer meniscus. And subsequently, the so-induced significant capillary forces drive the flow of colloidal suspension preferably toward this end and then cause unidirectional movement^25^. The results shown in this article are all examples of unidirectional movement from right to left in order not to be confused.

Figure 1(a) also shows that crystal region with stable mono green formed first on the left side of the tube, while the rest is multi-colored. Then the mono green region kept to extend to the right side until the whole tube was completely replaced at the end of the drying process. There is no obvious interface between mono-green and multi-colored region, but a color-blurred transition region. To investigate the crystal structures, seven points with equal distance were selected to collect reflection spectra, as shown in Fig. 1(b). Because wave vector can be written as $q=(4\pi n/\lambda )sin(\theta /2)$, where *n* is the refractive index of the suspension, *λ* is the vacuum wavelength, and *θ* is the scattering angle (*θ* = 180°), the reflection spectra can be converted to intensity distribution at different wave vectors. By analyzing the relative positions of the diffraction peaks originating from different crystal planes, the crystal structure can be determined^29^. Taking point *g* in Fig. 1(b) as an example, the wave vector corresponding to diffraction peaks are 21.36, 24.75 *μm*^−1^ and the ratio is $\sqrt{3}/\sqrt{4}$. According to ${q}_{hkl}=\frac{2\pi }{a}\sqrt{{h}^{2}+{k}^{2}+{l}^{2}}$ where *h, k, l* are the Miller indices, the crystal structure is fcc and the corresponding crystal planes are (111) plane and (200) plane. A more detailed description about the determination of the crystal structure from reflection spectra can be found in ref. ^30^. By the same way, the crystal structures at all the other points can also be determined to be fcc. The corresponding crystal planes of each peak are indicated in Fig. 1(b). The selected region includes a transition region between the close packed region (point *a* with the volume fraction of 73%) on the left side and initially concentration region (point *g* with the volume fraction of 11%) on the right side, as mentioned above. In this region the concentration increases continuously from right to left until its maximum allowable value (the close packed), so we can call it concentrated region. Actually, in this region all possible volume fractions of colloidal suspension inside the tube should be covered. Please note that in the enlarged graph in Fig. 1(b), we can see the color varies radially, which is due to the curved tube wall. In colloidal crystallization, the close-packed surface fcc (111) tend to arrange parallel with the tube wall, as a result the local orientations of fcc (111) will change accordingly to be perpendicular to the radial directions. Therefore, for a fixed viewing direction, the color looks different.

Volume fraction in the crystal region can be evaluated by reflection spectra if the crystal structure is known. In this study, the crystal structures of different samples are all fcc during drying process. Therefore, the volume fraction (*ϕ*) can be estimated as^31^:

1 $$\phi =\frac{2\pi }{9\sqrt{3}}{\left(\frac{2nd}{\lambda }\right)}^{3}$$

where *n* is the volume-weighted average of the refractive indices which relates with the refractive indices of particle and the medium which can be estimated by $n={n}_{particle}\phi +{n}_{water}(1-\phi )$, *d* is the particle diameter, *λ* is the wavelength of first order reflection peak from (111) plane.

**Figure 2:**
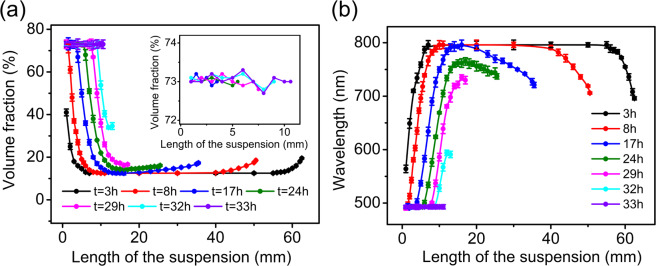
Changing tendency of volume fraction distribution (**a**) and corresponding wavelength distribution (**b**) in the suspension during drying process. The inset of Fig. 2(a) is an enlargement of the close packed region. In order to present the close packed region more clearly, the error bars are removed and only the data of close packed region are shown.

Figure 2 presents plots for both volume fraction distribution (a) and corresponding wavelength distribution (b) at different drying intervals. The given volume fractions were calculated according to their respective corresponding wavelengths, recorded by reflection spectra, by using Eq. (1). In order to effectively analyze the results, we assume that the abscissa axis is along the direction of the suspension denoted by *L* and assign its positive direction to be from left to right, while still keeping volume fraction (*ϕ*) being the vertical axis.

Except in the stage near the end of the evaporation process, these ($\phi -L$) curves share a common feature, that is, their middle segments are low-lying, rising at both ends, although the left side rises up much more than the right side. This feature implies that evaporation takes place in two directions of the tube, though overwhelmingly stronger in one direction than the other.

Now, we will take the curve at *t* = 17 h as an example to discuss why the suspension in drying process should be divided into four regions:

#### Close packed region

This region corresponds to the leftmost segment of the curve. The segment is a flat straight line with a gradient $d\phi /dL=0$ so its volume fraction keeps a constant value ($\phi =73 \%$). As evaporation proceeds, this segment continues to extend to the right until it annexes all the rest at the end of evaporation.

#### Concentrated region

This region is next to the right side of the close packed region. Its corresponding $\phi -L$ segment is an oblique line with a gradient $\,d\phi /dL < 0$. That means with the decrease of *L*, the concentration is increasing, namely, a concentration (from 11% to 73%) from right to left (note: the positive direction of *L* is from left to right).

#### Initial concentration region

This region is next to the right side of the concentrated region mentioned above. The segment is also a flat straight line with a gradient $\,d\phi /dL=0$, i.e., its volume fraction keeps a constant value and equals to the value for the initial state, that is $\,\phi =11 \%$.

#### Dilution region

This region corresponds to the rightmost segment of the curve. Its corresponding $\phi -L$ segment is an oblique line with a gradient $\,d\phi /dL > 0$. That means with the decrease of *L*, the concentration decreases, namely, a dilution (from 19% to 11%) from right to left to relieve (through diffusion). The increased concentration ($\phi =19 \%$) at the right end of the tube is due to evaporation.

Now to sum up, the whole evaporation process can be described briefly as following: Before *t* = 3 h, there is no close packed crystals formed because the volume fraction on the left side is not high enough. Until about *t* = 8 h, the close packed crystals ($\phi =73 \%$) started to emerge. There were four distinct regions appeared in the suspension from *t* = 8 h to *t* = 17 h in the drying process. In the process, the close packed region is expanding, while the rest three regions are shortening. Eventually, only close packed region existed after *t* = 33 h.

**Figure 3:**
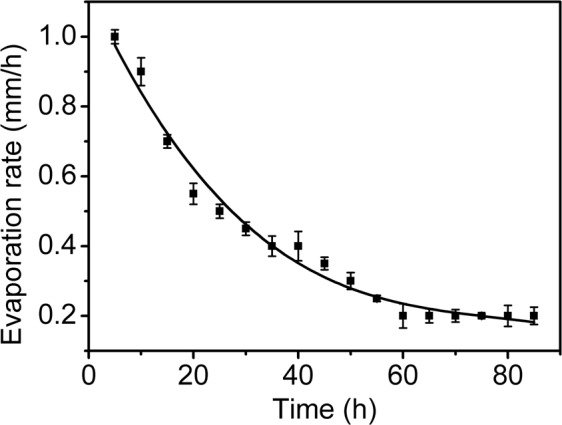
The change of evaporation rate of suspension on the right side of the tube.

Is there any evaporation at the right end of the suspension in the capillary tube? To solve this issue, we carried out an evaporation experiment with the same capillary tube but sealed at left end under exactly the same experimental conditions. As shown in Fig. 3, the evaporation rate decreases rapidly with time at the early stage of evaporation, while at the late stage the evaporation rate decreases slowly and approaches a stable value. The result indicates that the movement of the liquid-air interface can cause a decrease of evaporation rate, but not to be zero.

### Drying process of initially disordered suspension (B)

**Figure 4:**
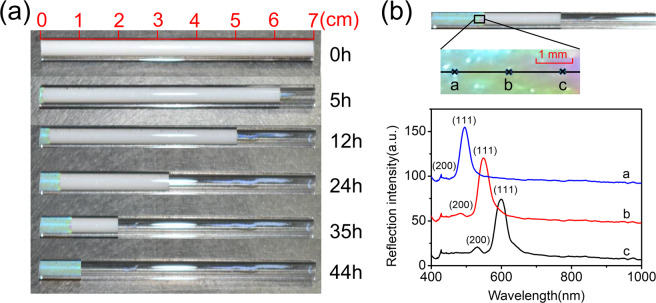
(**a**) Photographs of initially disordered suspension during drying process in chronological order. (**b**) Enlarged graph of the concentrated region with an approximate size 3 × 1 *mm*^2^ (*a*-*c* represents the measure points with equal distance) and the corresponding reflection spectra (The spectra were shifted for clarity).

As a more general case and also for comparison with others’ results, we also investigated the initially disordered suspension (B), similar to ref. ^25^. First, we will show what we observed when the concentration measurement was not involved. Figure 4(a) displays the phenomenon and feature that we found during drying process, similar to Fig. 1(a), i.e., mono green color appeared on the left side of the tube, and also the unidirectional movement of suspension from right to left.

Comparing Fig. 1(a) with Fig. 4(a), we can see that the evaporation rate for initially ordered suspension is higher than that for the disordered one. What is the cause of this phenomenon? There is little doubt that the gaps between particles provide transporting channels for water evaporation. These channels are less obstructed for water going through in the ordered structure than in the disordered structure and therefore an ordered suspension has higher drying rate.

The right half for suspension (B) exhibited milky white instead of colorful suspension because the suspension was initially in disordered state. Since there was obvious boundary between left and right side, dividing the colloidal suspension into two regions seems quite reasonable^25,28^. However, more detailed information provided by the real-time measurement of local concentration gave us new insight into the problem.

Our findings presented for initially ordered suspension (A) suggested us to pay special attention to the boundary between the two regions. So in the adjacent region across the interface of the two phases, we choose three representative observation points (from *a* to *c*) with equal distance as shown in Fig. 4(b). Once again, our reflection spectra method can be used to determine local concentrations only suitable for ordered structures. There are no concentration data available except for the region with ordered structures. The corresponding reflection spectra indicate that the colloidal structures on left side were all fcc.

**Figure 5:**
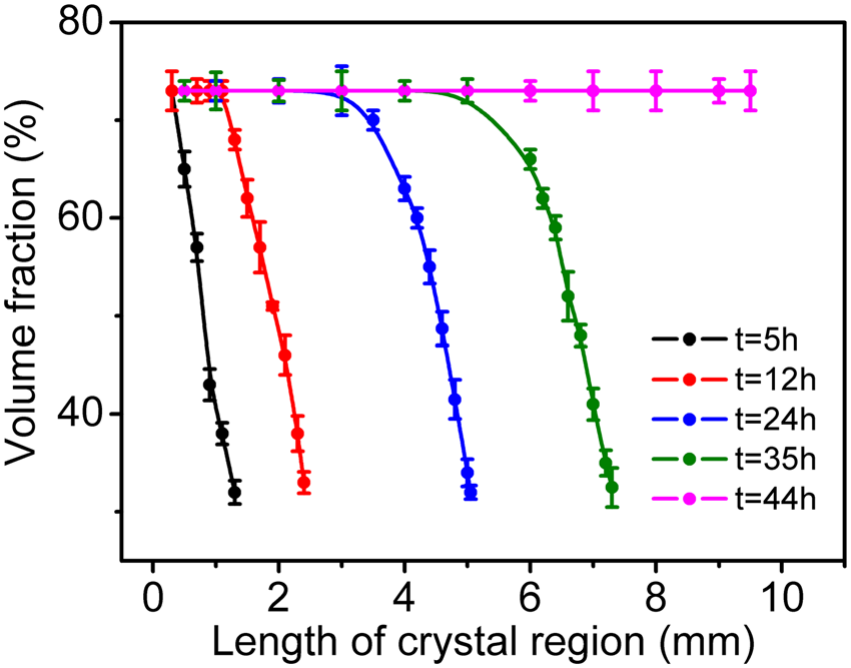
Volume fraction distributions in the crystal region during drying process.

The detailed volume fraction distributions of crystal region during drying process are presented in chronological order in Fig. 5. At *t* = 5 h, there is a change in volume fraction from 73% (at left terminal) to 32% (at the crystal-liquid interface), indicating the existence of a concentrated region with a gradient $\,d\phi /dL < 0$. From *t* = 12 h to *t* = 35 h, such a range of volume fraction in the concentrated region kept unchanged and close packed region ($\phi =73 \%$) grown gradually from left to right. At *t* = 44 h, all the suspension changed into close packed crystal ($\phi =73 \%$) and the concentrated region disappeared.

From Fig. 5, the close packed region ($\phi =73 \%$) and the concentrated region ($\phi =32 \% \,{\rm{to}}\,73 \%$) on the left side of the tube also existed, that is similar to initially ordered suspension. However, volume fraction of 32% at the crystal-liquid interface is larger than the initial volume fraction of 9%, which means the particle concentration in the disordered part is not homogeneous. More important reason for this is that our method has lost its measurement capability for lower concentrations of disordered suspensions (below $\,\phi =32 \%$). According to the results for initially ordered suspension (A) discussed in the previous section, we can reasonably speculate that in the concentrated region there should be a volume fraction change ranging from 9% to 73%. In the same way, we can also conjecture that there should be an initial concentration region with $\phi =9 \%$ and the gradient $d\phi /dL=0$ of the $\phi -L$ curve; and a dilution region with a gradient $d\phi /dL > 0$ of the $\phi -L$ curve though we are unable to identify the exact value of $\phi$ at the right end of the suspension.

### Model of drying process of colloidal suspension in tube

**Figure 6:**
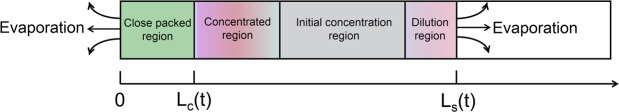
Model of drying process of colloidal suspension in tube.

In order to understand the drying process, we further put forward a new model of drying process of colloidal suspension considering the coupling effect of evaporation, diffusion and convection, which is presented in Fig. 6. Based on our observation and discussion in the previous section, we set up four regions inside the tube and the water evaporates from two sides. Here, the drying process is treated as one-dimensional model because the radial dimension of the tube is only 2% of its axial dimension. In addition, apart from the interface between the suspension and the tube wall, there is no other physical factor that can cause the radial variation of the concentration. For colloidal suspension drying in capillary tube, theoretically, the concentration profile can be quantified by the following one-dimensional transport equation^32^:

2 $$\frac{\partial \phi }{\partial t}+{V}_{1}\frac{\partial \phi }{\partial x}=\frac{\partial }{\partial x}(D(\phi )\frac{\partial \phi }{\partial x})$$

3 $${L}_{s}(t)={L}_{0}-({V}_{1}+{V}_{2})t$$

where *V*_1_ is the evaporation rate of suspension on the left side; *V*_2_ is the evaporation rate of suspension on the right side. The convection term is mainly caused by evaporation on the left. $D(\phi )$ is the collective diffusion coefficient which depends on the particles volume fraction, and the expression according to Stokes-Einstein equation is $D(\phi )={k}_{B}T/6\pi \eta r$, the viscosity term based on Einstein viscosity law is expressed as $\eta ={\eta }_{0}(1+2.5\phi )$, *k*_*B*_ is the Boltzmann constant, *T* is the absolute temperature, ${\eta }_{0}$ is the solvent viscosity. ${L}_{s}(t)$ is the length of suspension during drying process and *L*_0_ is the initial suspension length. Boundary conditions on transport equation are that the flux of particles at the liquid-air interface matches the recession due to evaporation, hence^33^:

4 $${\left(D(\phi )\frac{\partial \phi }{\partial x}\right)}_{x={L}_{c}(t)}=-{V}_{1}{\phi }_{x={L}_{c}(t)}\,{\rm{and}}\,{\left(D(\phi )\frac{\partial \phi }{\partial x}\right)}_{x={L}_{s}(t)}={V}_{2}{\phi }_{x={L}_{s}(t)}$$

where *L*_*c*_(*t*) is the length of close packed region during drying process.

**Figure 7:**
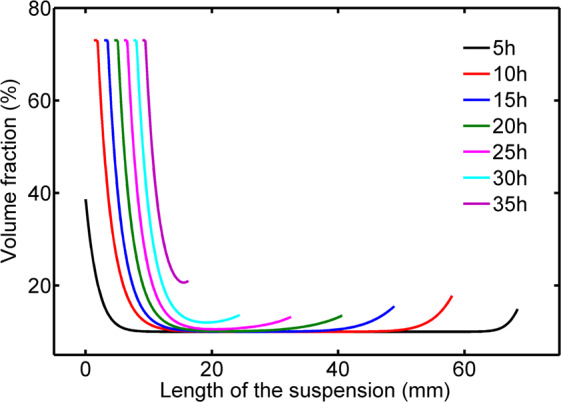
The calculated concentration profile of colloidal suspension during drying process.

Equations (2) to (4) are resolved numerically by finite difference method using the boundary conditions and the initial concentration of the suspension. Curves of $\phi -L$ in chronological order in Fig. 7 demonstrate that the numerical solution of our model are well consistent with the basic features, shown in Fig. 2, obtained from our experiments.

### Formula of estimating the concentration of close packed region

As discussed above, our study shows that the suspensions, whether they are initially ordered or disordered, would finally have the same structure (close packed type) with volume fraction of 73%. A formula for estimating the concentration of close packed region, based on previous model of two regions, is presented as^25^:

5 $$({\phi }_{d}-{\phi }_{0})\frac{d({L}_{c})}{dt}={\phi }_{0}\frac{d({L}_{s})}{dt}$$

where ${\phi }_{d}$ is the volume fraction of the close packed region, ${\phi }_{0}$ is that of initial colloidal suspension, $d({L}_{c})/dt$ is the moving rate of close packed front and $d({L}_{s})/dt$ is that of liquid-gas interface. However, Eq. (5) is not applicable for all cases, e.g., the evaporation rates change with time. Besides, it is not easy to accurately measure the values of moving rates, making it difficult to determine the concentration of close packed region. The final concentration of close packed region is a useful physical quantity for investigating drying process, especially for the dynamic of drying based on Darcy’s law^34^. Based on our model dividing the suspension into four regions, we have the following simple formula to accurately calculate the concentration of close packed region

6 $${\phi }_{d}{L}_{c}={\phi }_{0}{L}_{0}$$

where *L*_*c*_ is the final length of close packed region and *L*_0_ is the length of initial colloidal suspension. In the final state, the length of close packed region is about 10.5 mm, the volume fraction of close packed region can be easily estimated using Eq. (6), $\,{\phi }_{d}=73.3 \%$, which is consistent with that calculated by reflection spectra.

## Conclusion

Benefiting from its non-invasive, real-time and *in-situ* detecting ability, reflection spectrometer was adapted to access the information essential to understand process of the drying of colloidal suspension inside the two-end open tube in this study. Our experiments showed that particles continuously migrated and accumulated toward one end in the drying process with retraction of the suspension column at the other end, resulting in formation and growth of close-packed colloidal crystals at the end region. We found that evaporation took place in two directions of the tube, though much stronger in one direction than the other; during drying process, colloidal suspension column along the tube could be divided into four regions with different characteristics. We further proposed a model to formulate the evolution of concentration profile, which was consistent with the experimental results. In addition, according to solute conservation, we presented a simple way to estimate the concentration of close packed region.

## Materials and Methods

### Materials

The negatively charged polystyrene particles were purchased from Hugebio Inc, China. Before preparation, the original colloidal suspension was centrifuged and washed three times with deionized water, and then stored at 4 °C before the experiment. Other chemical reagents employed in this study, including Octadecyltrichlorosilane (OTS) and chloroform, were all of analytical grade and purchased from Sinopharm Chemical Reagent Co., Ltd, China. OTS chloroform solution with a concentration of 4 mM was prepared by dissolving OTS in chloroform and stored at 25 °C before the experiment.

### Sample preparation and experimental methods

Two kinds of suspensions, A and B, were investigated in this study. The difference between them is their initial state. The initial state for A and B are ordered suspension (particles in crystalline state) and disordered ones (particles in amorphous state), respectively. The suspensions were prepared by diluting the centrifuged latex. Volume fractions are 11% for A (deionized by ion-exchange resin) and 9% for B, respectively.

To avoid particles adhering to the inner wall of the capillary tubes, the capillary tubes were treated with hydrophobicity. The circular quartz capillary tubes (diameter: 1.5 mm, length: 70 mm) with both sides open were washed in water three time by ultrasonic with 150 W power and 40 kHz frequency and then dried in the drying oven at 100 °C for 1 hour. Subsequently the capillary tubes were immersed in OTS chloroform solution for 30 minutes and then dried in the drying oven at 180 °C for 2 hours.

The prepared samples were put into the capillary tubes, and then the tubes were put horizontally on the optical platform to avoid external vibration. The samples were evaporated naturally at room temperature.

### Characterization of crystal structure

The changes of crystal structures and particle concentrations along the long axis of the capillary tubes were measured by reflection spectra, which is the same as that described in our previous study^30,35^. The optical fiber head of the reflection spectrometer, which can be adjusted in three dimensions with displacement precision of 0.05 mm, was oriented perpendicular to the long axis of the capillary tube.

### Characterization of particles diameter

**Figure 8:**
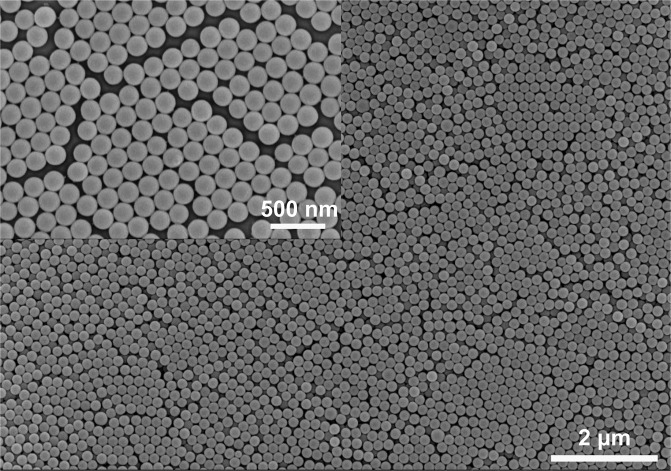
SEM image of the used colloidal particles.

The mean particles diameter is a vital parameter needed for calculating the concentration of the colloidal suspension in the current study. To make its data more reliable, we adopted scanning electron microscope (SEM, Hitachi S-4800) to determine particles diameter and the statistical mean diameter so obtained is $197\pm 1$ nm (Fig. 8). SEM was operated at an accelerating voltage of 10 kV and emission current of 10 μA, two kinds of magnification are 10000 and 40000, respectively.

### Characterization of evaporation rate

The movement of liquid-gas interface and close packed front was recorded by the digital camera (Nikon D5600). Specifications, resolution and zoom of captured images are 6000 × 4000, 300 dpi and 42 mm. The images were captured every hour. By measuring the positions of the liquid-gas interface, we can obtain the displacement of the interface versus time. Taking the derivative of the displacement with time, we obtain evaporation rate versus time.

## Data Availability

The datasets generated and analyzed during the current study are available from the corresponding author on reasonable request.
